# Analysis of Incidentally Diagnosed Patients with Coronavirus Disease 2019 at the Emergency Department: Single-Center Clinical Experience

**DOI:** 10.5152/eurasianjmed.2021.20291

**Published:** 2021-06

**Authors:** Ali Gur, Erdal Tekin, Ibrahim Ozlu

**Affiliations:** Department of Emergency, Atatürk University School of Medicine, Erzurum, Turkey

**Keywords:** COVID-19, Infection, Emergency Department, Incidental COVID-19

## Abstract

**Objective:**

Asymptomatic patients with coronavirus disease 2019 can present with signs of various diseases to hospitals. We aimed to present patients who presented to the emergency department without any coronavirus disease 2019 symptoms and were incidentally diagnosed with coronavirus disease 2019 in addition to the diagnosis related to their complaints on presentation.

**Materials and Methods:**

The study included patients presented to the emergency department of a hospital in Erzurum (Turkey) with non-coronavirus disease 2019 symptoms and were incidentally diagnosed with coronavirus disease 2019 on the basis of their chest computed tomography findings. The patients’ primary diagnoses were evaluated, and a reverse transcriptase-polymerase chain reaction was performed to confirm the incidental coronavirus disease 2019 diagnoses.

**Results:**

The 42 patients included in the study had various complaints, and the most common complaint was abdominal pain in 8 patients (19%). The other complaints were at different rates. The most common diagnose was ST-elevated myocardial infarction in 6 patients (14.3%), and the other diagnoses were rib fracture in 3 patients (7.1%) and other similar diseases in the remaining patients.

**Conclusion:**

Patients with highly the contagious coronavirus disease 2019 can sometimes be asymptomatic and can be incidentally diagnosed with coronavirus disease 2019 after presenting to emergency departments with symptoms and manifestations other than those of coronavirus disease 2019. Therefore, healthcare professionals working in the emergency department should approach all patients who present to the emergency service as potential coronavirus disease 2019 carriers and wear their protective equipment and take necessary precautions.

## Introduction

The epidemic novel coronavirus disease 2019 (COVID-19) is an infectious disease that first appeared in Wuhan, China in November 2019 and then spread to the whole world.^1^ COVID-19 is rapidly transmitted from person to person, and people are very sensitive and susceptible to this disease.^2^ In Turkey, the first case was reported on March 11, 2020, and since then, 205,758 patients have been diagnosed with the disease.^3^

Individuals with COVID-19 can show symptoms or transmit the virus freely in the community asymptomatically. These patients can present to hospitals with any symptom that may occur under normal conditions. However, because they are asymptomatic, they can easily transmit the disease to healthcare personnel and other people around them.^4^ Many asymptomatic cases have been published in case reports in different journals.^5^–^8^ These publications generally discuss the asymptomatic transmission rate, rate of spread, radiological findings, epidemiological features of asymptomatic patients, and precautions to be taken for isolation purposes.

In this study, we aimed to present the analysis of 42 patients who presented to the emergency department with any symptom other than those of COVID-19, who were incidentally diagnosed with COVID-19 in addition to the disease related to admission complaints, and who received treatment for both primary disease and COVID-19.

## Materials and Methods

### Study Design

The study included 42 patients presented to the emergency department of Ataturk University (Erzurum, Turkey). Approximately 250–300 patients apply to the emergency department of Ataturk University daily. In our study, the patients were identified between June 1, 2020 and June 31, 2020. Our study consists of patients who presented to the emergency department with any symptoms other than those of COVID-19 and for whom incidental findings of COVID-19 were detected on chest computed tomography (CT). These patients were diagnosed with both COVID-19 and their primary disease. In our study, patients with a history of trauma, aged >18 years, without COVID-19 disease symptoms (such as fever, cough, and respiratory failure), with COVID-19 findings on tomography, and with any diagnosis other than COVID-19 in the emergency department were included. Patients aged > 18 years, pregnant women without COVID-19 findings on tomography, and patients that had COVID-19 symptoms (such as fever, cough, and respiratory failure) were excluded. For this study, ethics committee approval was received from Ataturk University Ethical Committee with approval number B.30.2.ATA.0.01.00/271 and was dated May 28, 2020.

The patients had no additional symptoms other than the complaints enumerated at presentation. However, differential diagnoses (e.g., aortic dissection, pulmonary embolism, and others) were considered in the detailed anamnesis and physical examination obtained from the patients. Therefore, chest CT was performed on the patients to exclude the main diagnosis from differential diagnoses. According to their primary complaints, patients were evaluated and were suspected to have COVID-19 on the basis of findings incidentally detected on the chest CT performed for their advanced diagnostic examination. Then, a nasopharyngeal swab sample was taken from these patients, and the diagnosis of COVID-19 was confirmed by the reverse-transcriptase polymerase chain reaction (RT-PCR) test. The patients were admitted to the COVID-19 services of the hospital reserved for the isolation of patients with COVID-19. In these services, the patients were followed up and treated by clinicians for both COVID-19 and their primary diseases. From the chest CT findings, the presence of ground-glass opacity (GGO), paving-stone appearance, and consolidation areas was considered significant for the diagnosis of COVID-19. The patients who had related chest CT findings but tested negative for RT-PCR test were also accepted to have COVID-19 and were treated accordingly.

### Data Collection

The patients’ epidemiological data included in the study are age, sex, complaints at presentation, intercity or international travel history, history of chronic diseases, and history of contact with patients with COVID-19, which were obtained from the patients or their relatives. Then, diagnoses made at the emergency department, vital signs, body system related to the patients’ complaints (e.g., gastrointestinal, musculoskeletal, and circulatory), CT findings, abnormal laboratory findings, treatments received for COVID-19, advanced procedures if applied, number of days on hospitalization, and mortality status were also recorded.

### Statistical Analysis

The Statistical Package for the Social Sciences software, version 22.0 (IBM SPSS Corp.; Armonk, NY, USA) was applied for all analyses. Continuous variables were expressed as means (standard deviations) or medians (interquartile range [IQR]) as appropriate. Categorical data were presented as numbers (percentages).

## Results

### Demographic and Epidemiological Characteristics

In this study, a total of 42 patients presenting to the emergency department were incidentally diagnosed with COVID-19. The patient demographic and epidemiological characteristics are presented in Table 1.

### Symptoms

The most common complaint at presentation to the emergency department was abdominal pain (*n* = 8, 19%); the other complaints are presented in Table 2.

### Vital Signs

The vital signs of the patients were as follows: mean systolic blood pressure of 130 ± 24.6 (minimum-maximum = 80–89) mmHg, mean diastolic blood pressure of 84 ± 17 (minimum-maximum = 42–135) mmHg, body temperature of 36.3 ± 0.1°C (minimum-maximum = 36.1–36.7°C), mean respiratory rate of 20 ± 3 (minimum-maximum = 16–26) breaths per minute, mean oxygen saturation of 94 ± 3% (minimum-maximum = 82–98%), and mean pulse rate of 77 ± 19 (minimum-maximum = 60–180) beats per minute.

### Non-Coronavirus Disease 2019 Diagnoses

In addition to the incidental diagnosis of COVID-19, at the emergency department, the most common diagnose was ST-elevated myocardial infarction (STEMI) in 6 patients (14.3%), and the other diagnoses are presented in Table 2.

### Chest Computed Tomography and Reverse Transcriptase-Polymerase Chain Reaction Characteristics

All patients underwent a chest CT for diagnostic purposes on the basis of their clinical presentation. The CT findings of the 42 patients are presented in Table 3. Of the patients evaluated to have COVID-19 on the basis of the chest CT findings, 34 (80.9%) tested positive, and 8 (19.1%) tested negative on RT-PCR. None of the patients with positive RT-PCR tests also had symptoms of COVID-19.

### Laboratory Findings

When the laboratory parameters of the diagnosed patients were evaluated, increased values were observed for the white blood cell value in 19 patients (43.2%), neutrophils in 18 patients (42.9%), C-reactive protein (CRP) value in 18 patients (42.2%), D-dimer in 11 patients (26.2%), lactate in 11 patients (26.2%), aspartate aminotransferase (AST) in 6 patients (14.3%), alanine aminotransferase (ALT) in 6 patients (14.3%), troponin I in 7 patients (16.7%), creatinine in 1 patient (2.4%), carboxyhemoglobin (COHb) in 1 patient (2.4%), amylase in 1 patient (2.4%), glucose in 1 patient (2.4%), and total bilirubin in 1 patient (2.4%), whereas decreased values were seen in 5 patients (11.9%) for lymphocyte, 4 patients (9.5%) for hemoglobulin, and 1 patient (2.4) for glucose.

### Treatment and Hospitalization

All the patients were hospitalized for both COVID-19 and the primary disease treatment. The median length of hospital stay was 12 days (IQR = 1–21 days). For treatment of COVID-19, hydroxychloroquine (Plaquenil) (200 mg peroral, twice a day) and levofloxacin (500 mg intravenous, once a day) were administered to 41 patients (97.6%). In comparison, the remaining 1 patient (2.4%) received favipiravir (200 mg peroral, twice a day) together with hydroxychloroquine (200 mg peroral, twice a day). The hospitalized patients were also given additional appropriate medical treatment for their primary diseases. Besides, surgery was performed in 3 patients (7.1%), percutaneous transluminal coronary angiography was performed in 6 patients (14.3%), hemodialysis was performed in 1 patient (2.4%), and oral endoscopy treatment was performed in 2 patients (4.8%). Of the patients receiving treatment for COVID-19 and primary disease, 4 (9.5%) died, and the other 38 patients (90.5%) were discharged from the hospital with full recovery. No symptoms of COVID-19 (fever, cough, dyspnea) were observed in the discharged patients during their hospitalization.

## Discussion

COVID-19 is a disease that has spread worldwide and infected millions of people. It is known that COVID-19 is more infectious than severe acute respiratory syndrome and Middle Eastern respiratory syndrome.^9^ It is also reported that the ratio of COVID-19 infection to asymptomatic cases ranges from 17.9% to 30.8%.^10^,^11^ Asymptomatic COVID-19 carriers can apply to the emergency department with complaints of any other disease. In this study, we reported 42 patients admitted to the emergency department with symptoms other than those of COVID-19 and were incidentally diagnosed with this disease.

Studies have shown that the risk of transmission in COVID-19 is greater among those with chronic diseases and elderly patients than among the younger age group.^12^ In this study, the median age of patients in whom COVID-19 was incidentally detected was 62 (IQR = 20–88) years, and 23 patients (55%) had a history of chronic disease.

Although fever and cough are the most common symptoms of COVID-19, respiratory failure, diarrhea, myalgia, and anosmia can also be seen or patients may be asymptomatic.^13^ In our study, none of the COVID-19 symptoms was present in any of our patients. Our patients presented to the emergency room with symptoms associated with other diseases and conditions, such as post-traumatic symptoms associated with falls and traffic accidents, abdominal pain, palpitations, chest pain, syncope, leg pain, bloody vomiting, and seizures.

The clinical features of COVID-19 are helpful in the diagnosis of the disease. However, apart from the clinical features currently defined, patients may also have different clinical manifestations. The literature contains case reports on COVID-19 detected after trauma or incidentally diagnosed COVID-19 in asymptomatic patients.^5^–^8^ Our sample consisted of patients who presented with non-COVID-19 symptoms and were diagnosed with various primary diseases, including ileus, stomach perforation, musculoskeletal disorders, STEMI, gastrointestinal system bleeding, and acute renal failure (ARF), hypoglycemic coma, intracranial hemorrhage, and central vein occlusion.

Chest CT is a beneficial method for showing viral pneumonia, and it is particularly beneficial in identifying COVID-19 cases. Although the role of chest CT in the diagnosis of COVID-19 remains controversial, the specific findings of the disease have been reported in articles published in the literature. GGO and consolidation areas on the chest CT are 2 main indications for COVID-19.^14^,^15^ In this study, the incidental detection of GGO and consolidation areas on chest CT while investigating our patients’ diagnoses related to their current complaints led us to suspect COVID-19. We took a nasopharyngeal swab sample from these patients and performed RT-PCR to confirm this suspicion. As a result, 34 of the 42 patients tested positive.

Leukopenia and lymphocytopenia are generally seen in laboratory findings of patients with COVID-19. AST is often increased. In infected patients, the hypersensitive troponin I value can be elevated. Inflammation markers, creatinine level, and prothrombin time can also be increased.^14^,^16^ In our study, when COVID-19 and other existing conditions were evaluated together, it was observed that lymphocytopenia coexisted in 5 patients (11.9%). Furthermore, hemoglobulin and glucose levels were reduced in 4 (9.5%) and 1 (2.4%) patients, respectively. Finally, increased values were detected for AST in 6 patients (14.3%), CRP in 18 patients (42.9%), D-dimer in 11 patients (26.2%), lactate in 11 patients (26.2%), ALT in 6 patients (14.3%), troponin I in 7 patients (16.7%), creatinine in 1 patient (2.4%), COHb in 1 patient (2.4%), amylase in 1 patient (2.4%), glucose in 1 patient (2.4%), and total bilirubin in 1 patient (2.4%).

COVID-19 mainly occurs as a mild to moderate disease, with a mortality rate of 3.2%.^17^ In this study, the mortality rate was 9.5% (*n* = 4). This high mortality rate can be attributed to the presence of chronic diseases and a further mortality-associated diagnosis added to the existing diseases in the emergency department. Of the 4 patients who died, 2 had diabetes mellitus, 1 had hypertension, and 1 had hypertension plus coronary artery disease history. The emergency department diagnosis of these patients was ARF, STEMI, stomach perforation, and urinary sepsis.

In conclusion, patients with highly the contagious COVID-19 can sometimes be asymptomatic and can be diagnosed with COVID-19 incidentally after presenting to the emergency department with non-COVID-19 symptoms and manifestations. Therefore, healthcare professionals working in the emergency department should approach all patients as potential COVID-19 carriers, wear their protective equipment, and take the necessary precautions.

**Main Points**

- COVID-19 patients can be detected incidentally in the emergency department.
- COVID-19 disease can also be seen together with the main diagnoses of patients.
- Healthcare professionals working in the emergency department should approach all patients as potential COVID-19 carriers and take the necessary precautions.

## Figures and Tables

**Table 1:** Demographic and Epidemiological Characteristics

Characteristics	n (%)
Age, median (IQR)	62 (22–88)
Female	19 (45.2)
Male	23 (54.8)
History of contact	5 (11.9)
History of domestic travel	8 (19)
Health employee	2 (4.8)
HT	6 (14.3)
CAD	5 (11.9)
HT+CAD	1 (2.4)
Familial Mediterranean fever	1 (2.4)
Epilepsy	2 (4.8)
DM	7 (16.7)
DM+HT	1 (2.4)
Smoking	18 (42.9)

CAD: coronary artery disease; DM: diabetes mellitus; HT: hypertension; IQR: interquartile range.

**Table 2:** Symptoms and Non-COVID-19 Diagnoses

Symptoms	n (%)	Non-COVID-19 diagnoses	n (%)
Fall-related trauma	4 (9.5)	Clavicula fracture	1 (2.4)
Abdominal pain	8 (19)	Cholecystitis	2 (4.8)
Traffic accidents	5 (11.9)	Rib fracture	3 (7.1)
Nausea/vomiting	5 (11.9)	Mandibular fracture	1 (2.4)
Syncope	5 (11.9)	Ileus	2 (4.8)
Contractions	2 (4.8)	Acute renal failure	1 (2.4)
Chest pain	4 (9.5)	Femur fracture	1 (2.4)
Headache	3 (7.1)	Ischemic CVO	2 (4.8)
Bloody vomiting	2 (4.8)	FMF attack	1 (2.4)
Leg pain	1 (2.4)	Pelvic fracture	2 (4.8)
Palpitations	1 (2.4)	Epileptic seizure attack	2 (4.8)
Back pain	1 (2.4)	Carbon monoxide poisoning	1 (2.4)
Battery-related trauma	1 (2.4)	STEMI	6 (14.3)
–	–	Intracranial hemorrhage	1 (2.4)
–	–	Pulmonary embolism	1 (2.4)
–	–	Pancreatitis	2 (4.8)
–	–	peptic ulcer	1 (2.4)
–	–	Brain tumor	1 (2.4)
–	–	GIS bleeding	2 (4.8)
–	–	Deep vein thrombosis	1 (2.4)
–	–	Hyperosmolar non-ketotic syndrome	1 (2.4)
–	–	Ventricular tachycardia	1 (2.4)
–	–	Gastric perforation	1 (2.4)
–	–	Calcaneus fracture	1 (2.4)
–	–	Urinary sepsis	1 (2.4)
–	–	Appendicitis	1 (2.4)
–	–	Scapular fracture	1 (2.4)
–	–	Hypoglycemic coma	1 (2.4)

COVID-19: Coronavirus Disease 2019; CVO: Central Vein Occlusion; FMF: Familial Mediterranean Fever; GIS: Gastrointestinal System; STEMI: ST-elevated myocardial infarction.

**Table 3:** Chest CT Findings of Asymptomatic Patients

CT findings	n (%)
GGOs	21(50)
GGO + consolidation areas	13 (31)
Paving stone appearance	6 (14.2)
Consolidation areas	2 (4.8)

CT: Computed Tomography; GCO: Ground-Glass Opacity.
